# Dermal Fibroblast Migration and Proliferation Upon Wounding or Lipopolysaccharide Exposure is Mediated by Stathmin

**DOI:** 10.3389/fphar.2021.781282

**Published:** 2022-01-28

**Authors:** Ruiyan Cen, Liqun Wang, Yongqing He, Chenda Yue, Yang Tan, Lingfei Li, Xia Lei

**Affiliations:** Department of Dermatology, Daping Hospital, The Army Medical University, Chongqing, China

**Keywords:** stathmin, microtubule, migration, proliferation, lipopolysaccharide

## Abstract

The dermal fibroblast is a crucial executor involved in wound healing, and lipopolysaccharide is a key factor in initiating the migration and proliferation of the dermal fibroblasts, followed by wound healing. However, the underlying molecular mechanism is still unknown. In this study, we demonstrated that stathmin increased concomitantly with p38/MAPK pathway activation by lipopolysaccharide stimulation of the human dermal fibroblast (HDF), which induced microtubule (MT) depolymerization followed by increased HDF migration and proliferation. In contrast, the application of taxol, the small interfering RNA transfection of stathmin, or the application of the p38/MAPK inhibitor SB203580 suppressed MT depolymerization and HDF migration and proliferation. Additionally, the overexpression of a MKK6(Glu) mutant, which constitutively activated p38/MAPK, resulted in MT depolymerization and, subsequently, promoted HDF migration and proliferation. Our data reveal a crucial role of stathmin in HDF migration and proliferation. These findings will provide new targets and strategies for clinical interventions in wound healing.

## Introduction

Wound healing is an interactive and dynamic biological process involving an inflammation phase, a proliferation phase, and a tissue remodeling phase (Kaplani et al., 2018; Canedo-Dorantes and Canedo-Ayala, 2019). The dermal fibroblast is a critical executor during wound healing, and its migration and proliferation are essential and rate-limiting steps to repair wounds due to its central role in the formation of granulation tissue. If granulation tissue formation is dysfunction, wound healing may be delayed or wounds may not heal at all. Upon wounding, various cell types are activated by exogenous or endogenous stimuli to create conditions for wound healing (Singampalli et al., 2020; Shi et al., 2021). Although the lipopolysaccharide (LPS) exposure has been proven to be a key initial factor in wound healing and the dermal fibroblasts play an essential role in this process, the correlation between LPS exposure and the dermal fibroblast and their potential regulatory mechanisms remain largely unclear. Therefore, a comprehensive understanding of the biological processes of the dermal fibroblasts during wound healing is required.

The microtubule (MT), a crucial component of the cytoskeleton, is composed of *α*- and *β*-tubulin heterodimers (Kim and Matthew, 2007). The transition between the depolymerization and polymerization of tubulin is recognized as MT dynamics, which are regulated by destabilizing proteins (e.g., stathmin family) and stabilizing proteins (e.g., microtubule-associated protein family) (Walczak, 2000; Hu et al., 2010). MT dynamics determine the biological behavior of cells. Previous studies have indicated that MT depolymerization leads to increased epidermal or tumor cell migration and proliferation, whereas MT polymerization abolishes migration and proliferation (Garcin and Straube, 2019; Zhang et al., 2019; Seetharaman and Etienne-Manneville, 2020). However, MT stabilization also promotes carcinoma invasion (Yoon et al., 2005). Thus, a further mechanistic exploration of MT dynamics is needed to understand its role in cell migration and proliferation (Zhang et al., 2013; Yan et al., 2019).

Stathmin is a widely expressed and highly conserved cytosolic protein that belongs to the stathmin family (Sobel, 1991). Stathmin destabilizes MTs by both binding tubulin dimers as well as directly binding MTs and promoting depolymerization (Cassimeris, 2002; Al-Bassam and Chang, 2011). Cell proliferation involves the progression from interphase to mitotic phase. Cytoplasmic MT regulatory proteins, such as the stathmin family, play a significant role in cell cycle regulation (Rubin and Atweh, 2004). Recent studies indicated that stathmin expression levels were closely correlated with cell proliferation and migration, with enhanced stathmin expression observed in highly proliferating neuroblastoma cells or gastric cancer cells (Hasegawa et al., 2007; Shu et al., 2019). In addition, the involvement of signal transduction in regulating stathmin expression includes mitogen-activated protein kinases (MAPKs), microtubule affinity-regulating kinases, and protein kinase A (Feng et al., 2017; Esnault et al., 2020). Previous studies indicated that p38/MAPK activation regulated stathmin in cardiomyocytes (Hu et al., 2010) and that p38/MAPK activation affected gallbladder carcinoma cell and inflammatory cell proliferation and migration by regulating stathmin (Wang et al., 2016; Esnault et al., 2020). However, the effect and mechanism of stathmin in LPS-induced the dermal fibroblast migration and proliferation during wound healing are still poorly understood.

In this study, we aimed to explore the role of stathmin in the human dermal fibroblast (HDF) migration and proliferation during wound healing. We found that stathmin promotes HDF migration and proliferation under LPS stimulation, and the p38/MAPK pathway is a potential upstream kinase of stathmin-dependent MT depolymerization in response to LPS stimulation. Collectively, these findings provide a novel role for stathmin in promoting HDF migration and proliferation, indicating its therapeutic potential in the treatment of wound healing.

## Materials and Methods

### Ethics Statement

The Laboratory Animal Welfare and Ethics Committee of The Army Medical University reviewed and approved the animal study (approval number: AMUWEC2019509).

### *In vivo* Wound Model

For the animal experiment, 8- to 12-week-old male wild-type C57BL/6 J mice (*n* = 5) were anesthetized with intraperitoneal injection of sodium pentobarbital, and full-thickness skin wounds were constructed on the mid-dorsal skin with 5-mm disposable biopsy punches.

### Cell Culture

HDF was bought from American Type Culture Collection (Cat# PCS-201-012, cell type: fibroblast, origin: human skin) and cultured in Dulbecco’s modified Eagle’s medium (DMEM) supplemented with 10% fatal bovine serum at 37°C, 5% CO_2_ atmosphere (Gusarov et al., 2021). Lipopolysaccharide (Cat# L4391, *e. coli*. 0111: B4, Merck, Germany) was used to stimulate HDF *in vitro*. Taxol (1 μM, Cat# S1150, Selleck, United States) or SB203580 (5 μM, Cat# S1076, Selleck, United States) was used to the culture and pretreated at 37°C for 1 h before LPS stimulation.

### Cell Proliferation Assay

The Cell Counting Kit-8 (CCK-8; Cat# C0042, Beyotime, China) assay is based on WST-8 and was used to assess cell proliferation (Liu et al., 2020). The plate was preincubated for 24 h. After the CCK-8 solution was added to the 96 well plate, the plate was incubated for an additional 4 h. The absorbance was measured at 450 nm using a microplate reader (ELX800, BioTek). In addition, cell proliferation was assessed using a Edu-488 kit (Cat# C0071S, Beyotime, China), which is based on the incorporation of thymidine analogue Edu (5-ethynyl-2’-deoxyuridine) in the process of DNA synthesis, and Edu is labeled by Alexa Fluor 488 through subsequent click reaction to detect proliferation. After being subjected to the different treatments, cell proliferation was assessed by Edu staining and analyzed by a fluorescence microscope (IX75, Olympus, Japan). Edu staining (green) in cultured cells is shown in the representative images. Nuclei were stained with Hoechst 33342, which could stain DNA (blue).

### Scratch Wound Healing Assay

The monolayers of HDFs were incubated at 37°C for 2 h with mitomycin-C (5 μg/ml, Cat# S8146, Selleck, United States) to inhibit cell proliferation before wounded with a 1-ml plastic pipette tip and then washed with phosphate-buffered saline (PBS) to remove redundant cell debris. The wound healing course was observed with a light microscope (IX75, Olympus, Japan). Cell migration was defined as the wound healing rate (wound healing rate = the wound distance (0 h) (inches)—the wound distance (24 h) (inches)/the wound distance (0 h) (inches)), which was analyzed with NIH ImageJ software.

### Immunohistochemistry

The mouse skin tissues were cut at wound edges and fixed in 4% paraformaldehyde (Cat# P0099, Beyotime, China), embedded in paraffin, and sectioned. Sectioned wound tissues were deparaffinized and rehydrated. Antigen retrieval was performed by heating the sections in citrate buffer (pH 6.0) in a microwave at 600 W for 8 min. To perform immunohistochemistry staining for stathmin, wound tissues were incubated with stathmin rabbit mAb (1:100, Cat# 13655S, Cell Signaling Technology, United States) as the primary antibody at 4°C overnight. The tissues were then rinsed and incubated with a biotinylated secondary antibody (1:500, Cat# GB23303, Servicebio, China) and streptavidin-HRP (Cat#SP-9001, ZSGB-BIO, China). The color was developed using DAB peroxidase substrate (Cat# ZLI-9018, ZSGB-BIO, China) until an optimal color was observed.

### Recombinant Adenovirus Construction and Transduction

The adenovirus that activates MAPK kinase 6 (MKK6(Glu)), which precisely and stably activates p38/MAPK signaling, was produced by Shanghai Gene Chem Co., Ltd. (China) (Hu et al., 2010). CMV-null adenovirus was used as the negative control. The multiplicity of infection is thirty. After transfection of the HDF with adenoviruses for 48 h, the cells were used for experiments.

### Small Interfering RNA (siRNA) Transfection

For RNA interference, cells were transfected with siRNA targeted for stathmin (siSTMN) (sense (5’-3’) GCA​CGA​GAA​AGA​AGU​GCU​UTT, antisense (3’-5’) AAG​CAC​UUC​UUU​CUC​GUG​CTT) or the corresponding scramble siRNA (siNC) (sense (5’-3’) UUC​UCC​GAA​CGU​GUC​ACG​UTT, antisense (3’-5’) ACG​UGA​CAC​GUU​CGG​AGA​ATT) with GP-transfect Mate according to the manual (https://www.genepharma.com/public/upload/1554367091.pdf). The siRNAs and GP-transfect-Mate were ordered from Shanghai Gene Pharma Company (China).

### Western Blot Analysis

The mouse skin tissues and whole-cell extracts were prepared in RIPA lysis buffer for Western blotting (Cat# P0013, Beyotime, China) and centrifuged at 13700 × g for 15 min at 4°C. After obtained the supernatants, protein concentrations were detected using a Bradford Protein Quantification Kit (Cat# P0010, Beyotime, China). The protein samples were loaded and separated by SDS–PAGE (Cat# 1610183, Bio–Rad, United States) and then transferred to PVDF membranes (Cat# FFP26, Beyotime, China). Membranes were incubated overnight at 4°C with specific primary antibodies. Sequentially, membranes were incubated with secondary antibodies and visualized using a ChemiDoc XRS System (ChemiDoc, Bio–Rad, United States). Primary antibodies used for Western blotting were as follows: stathmin (Cat# 11157-1-AP, Proteintech, United States), PCNA (Cat# 13110S, Cell Signaling Technology, United States), p38 (Cat# 8690, Cell Signaling Technology, United States), phosphorylated p38 (p-P38, Cat# 9211S, Cell Signaling Technology, United States), and *β*-actin (Cat# 2148S, Cell Signaling Technology, United States).

### Immunofluorescence Staining of Microtubules

HDFs were cultured in 15-mm diameter confocal petri dishes (NEST, China) overnight. After treatments, cells were fixed with 4% paraformaldehyde for 20 min. Then the cells were washed twice with PBS before blocking in immunostaining blocking buffer (Cat# P0102, Beyotime, China) for 1 h. After incubation with rat anti-tubulin antibody (Cat# ab6160, Abcam, United Kingdom) diluted in 1% bovine serum albumin (BSA) in PBS overnight at 4°C, the dishes were washed with PBS for three times and then stained with Alexa Fluor 488 fluorescent secondary antibody (Cat# A32731, Invitrogen, United States) at 37°C for 1 h in the dark. Nuclei were counterstained with 4’,6-diamidino-2-phenylindole (DAPI) (Cat# C1005, Beyotime, China), and the plates were sealed with anti-fluorescence quenching solution (Cat# P0126, Beyotime, China) before imaging. The pictures were acquired using a Leica confocal microscope (Leica Microsystems, Wetzlar, Germany).

### Statistical Analysis

Statistical analysis was handled with GraphPad Prism 8 (GraphPad Software, California, United States). All results are shown as the mean ± SEM. Comparisons between two groups were analyzed using a two-tailed unpaired *t*-test. Statistical significance among three or more groups was determined by one-way analysis of variance (ANOVA) and followed by Tukey’s multiple comparison test. *p* < 0.05 was considered to be significant.

## Results

### Lipopolysaccharide Promotes the Migration and Proliferation of Human Dermal Fibroblast and Microtubule Depolymerization

**FIGURE 1:**
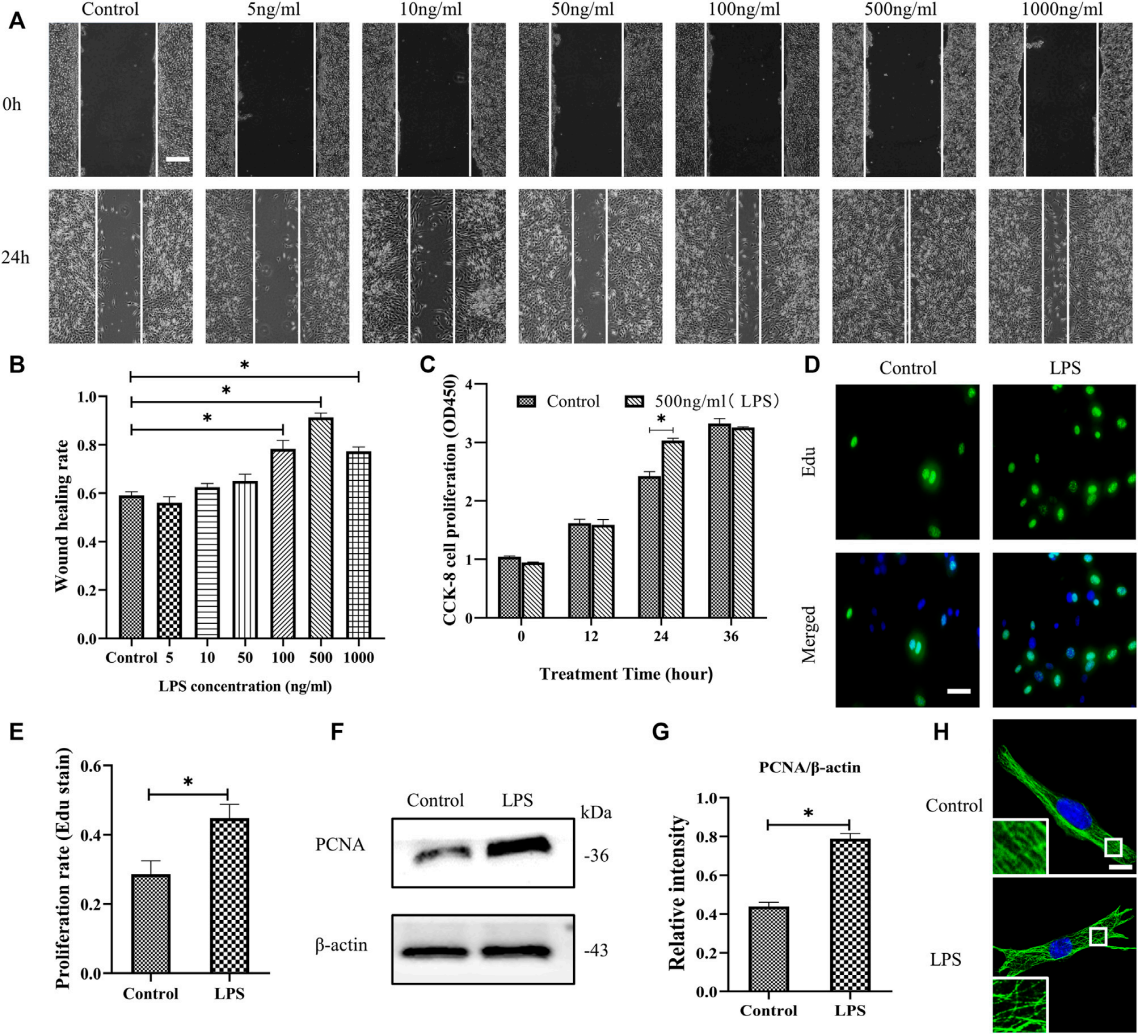
LPS promotes the migration and proliferation of HDF and MT depolymerization. **(A)** Scratch wound healing assays were performed to examine HDF migration with or without LPS (5, 10, 50, 100, 500 and 1,000 ng/ml) for 24 h. Representative pictures of the scratch wound are shown. Bar, 200 μm, and **(B)** the images were quantitatively analyzed (*n* = 5). **(C)** HDF was subjected to LPS (500 ng/ml) for 12, 24 and 36 h to detect cell proliferation using the CCK-8 assay (*n* = 6). **(D)** HDF proliferation was detected after 24 h with or without LPS by Edu staining and **(E)** quantitative analysis (*n* = 5). Nuclei were stained with Hoechst 33342. The merged image is to show the proportion of proliferating cells (green) to total cells (blue). Bar, 20 µm. **(F)** Western blotting was performed to detect PCNA with or without LPS (500 ng/ml) treatment and **(G)** quantitative analysis (*n* = 5). **(H)** Representative confocal tubulin immunofluorescence images showing the MT structure with or without LPS (500 ng/ml) for 24 h. The inserts show high-magnification images of the MT network. Bar, 10 µm. LPS, lipopolysaccharide; PCNA, proliferating cell nuclear antigen. The results are shown as means ± SEM. * *p* < 0.05 was considered to be significant.

The migration and proliferation of HDF are critical processes for wound repair. We used LPS to stimulate HDF *in vitro*. First, HDF was treated with six concentrations of LPS for 24 h to observe cell migration, which was determined using a scratch wound healing assay (Figures 1A,B). LPS in 500 ng/ml showed the most significant effect on the scratch wound healing assay. Therefore, we selected LPS (500 ng/ml) to examine HDF proliferation at 3 time points, as represented by CCK-8 kit testing. The results revealed that only at 24 h is LPS promoting proliferation compared to the control group (Figure 1C). According to the above results, LPS (500 ng/ml for 24 h) was used in the following experiments. The results showed that HDF proliferation was significantly increased about 18% by exposure to LPS, as shown by Edu staining (Figures 1D,E). PCNA, a cell proliferation marker, was also robustly increased (Figures 1F,G). In the morphological studies, the HDF subjected to LPS showed a less regular organization of MT network and some tubulin breakages that changed the MT appearance, whereas the control group revealed a uniformly distributed lattice network, as indicated by the tubulin immunofluorescence staining (Figure 1H).

### Involvement of Microtubule Depolymerization in Lipopolysaccharide-Induced Human Dermal Fibroblast Migration and Proliferation

**FIGURE 2:**
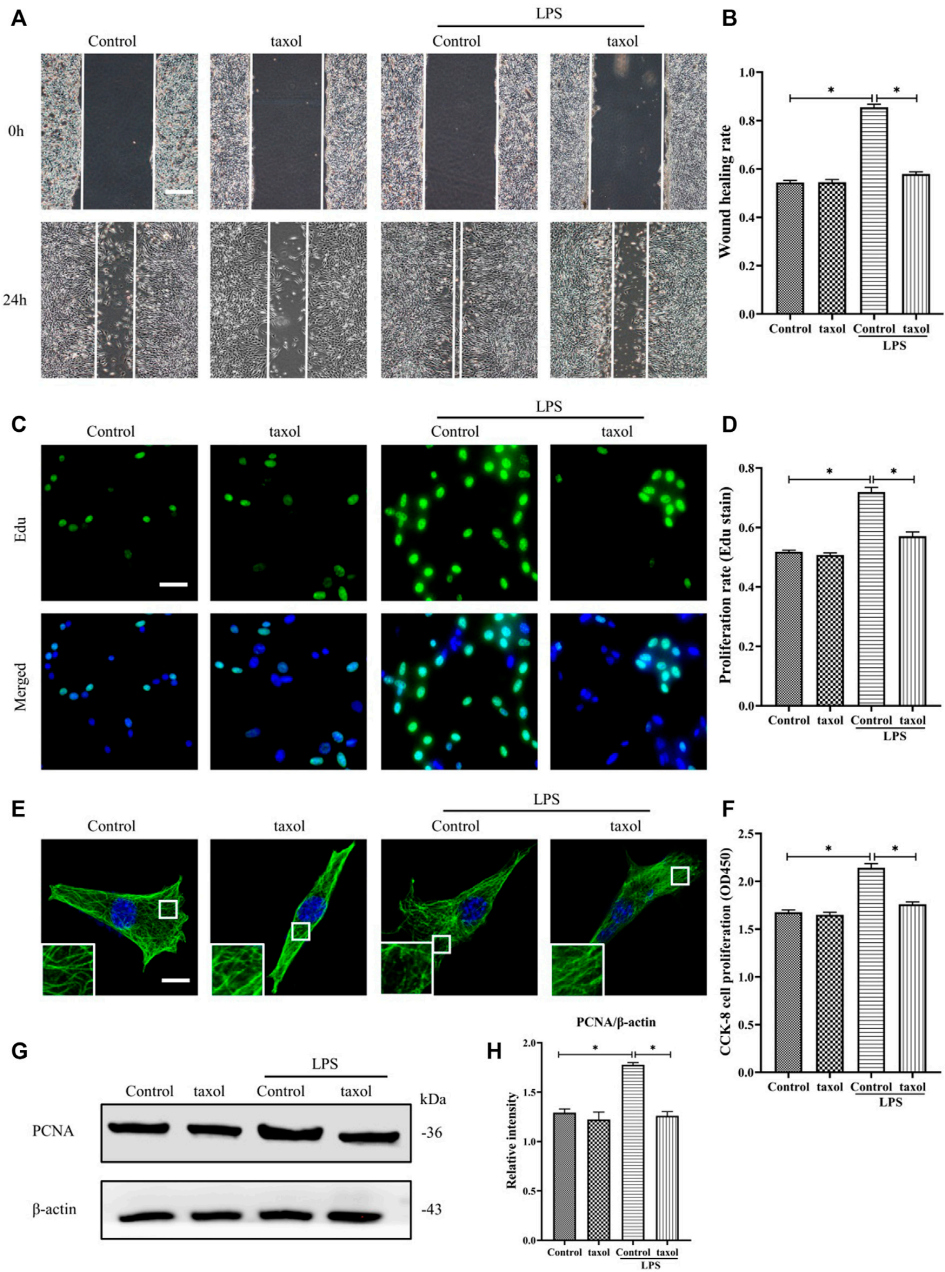
Involvement of MT depolymerization in LPS-induced HDF migration and proliferation. **(A)** Scratch wound healing assays were performed to detect HDF migration treated with LPS with or without taxol (1 µm) pretreatment, Bar, 200 μm, and **(B)** quantitative analysis (*n* = 5). **(C)** HDF proliferation was detected after LPS treatment with or without taxol pretreatment by Edu staining and **(D)** quantitative analysis (*n* = 5). Nuclei were stained with Hoechst 33342. The merged image is to show the proportion of proliferating cells (green) to total cells (blue). Bar, 20 µm. **(E)** For the tubulin immunofluorescence images of MTs, cells were pretreated with taxol before LPS stimulation. These inserts show high-magnification images of the peripheral MT network. Bar, 10 µm. **(F)** HDF was subjected to LPS with or without taxol pretreatment to detect cell proliferation using the CCK-8 assay (*n* = 10). **(G)** Western blotting was performed to detect PCNA after LPS treatment with or without taxol, and **(H)** the results were quantitatively analyzed (*n* = 5). The data are represented as the mean ± SEM. * *p* < 0.05 was considered to be significant.

In Figure 1H, it is shown that LPS induced MT depolymerization. To determine whether the observed HDF migration and proliferation are correlated with MT structural changes, taxol was used to stabilize the MTs and prevent them from depolymerizing. Our results showed that LPS stimulation promoted HDF migration and proliferation (Figures 2A–D,F–H); in contrast, taxol pretreatment significantly abrogated LPS-induced HDF migration, as reflected by the scratch wound healing assay (Figures 2A,B) and cell proliferation, as indicated by Edu staining (Figures 2C,D) and CCK-8 assay (Figure 2F). Moreover, the expression of the proliferation marker PCNA was also elevated after LPS challenge (Figures 2G,H). In contrast, taxol pretreatment suppressed this effect, while taxol group showed a change comparable to that of the control group (Figures 2G,H). Then, we assessed the role of an MT stabilizer in mediating LPS-induced MT depolymerization. The results showed that LPS induced MT depolymerization, whereas taxol pretreatment promoted the preservation of the MT network and led to an increased density of MT fragments near the plasma cell membrane after LPS challenge (Figure 2E). These findings suggested a crucial role of MTs in regulating LPS-promoted HDF migration and proliferation.

### Lipopolysaccharide-Induced Stathmin Expression and its Effects on Human Dermal Fibroblast Migration and Proliferation and Microtubule

**FIGURE 3:**
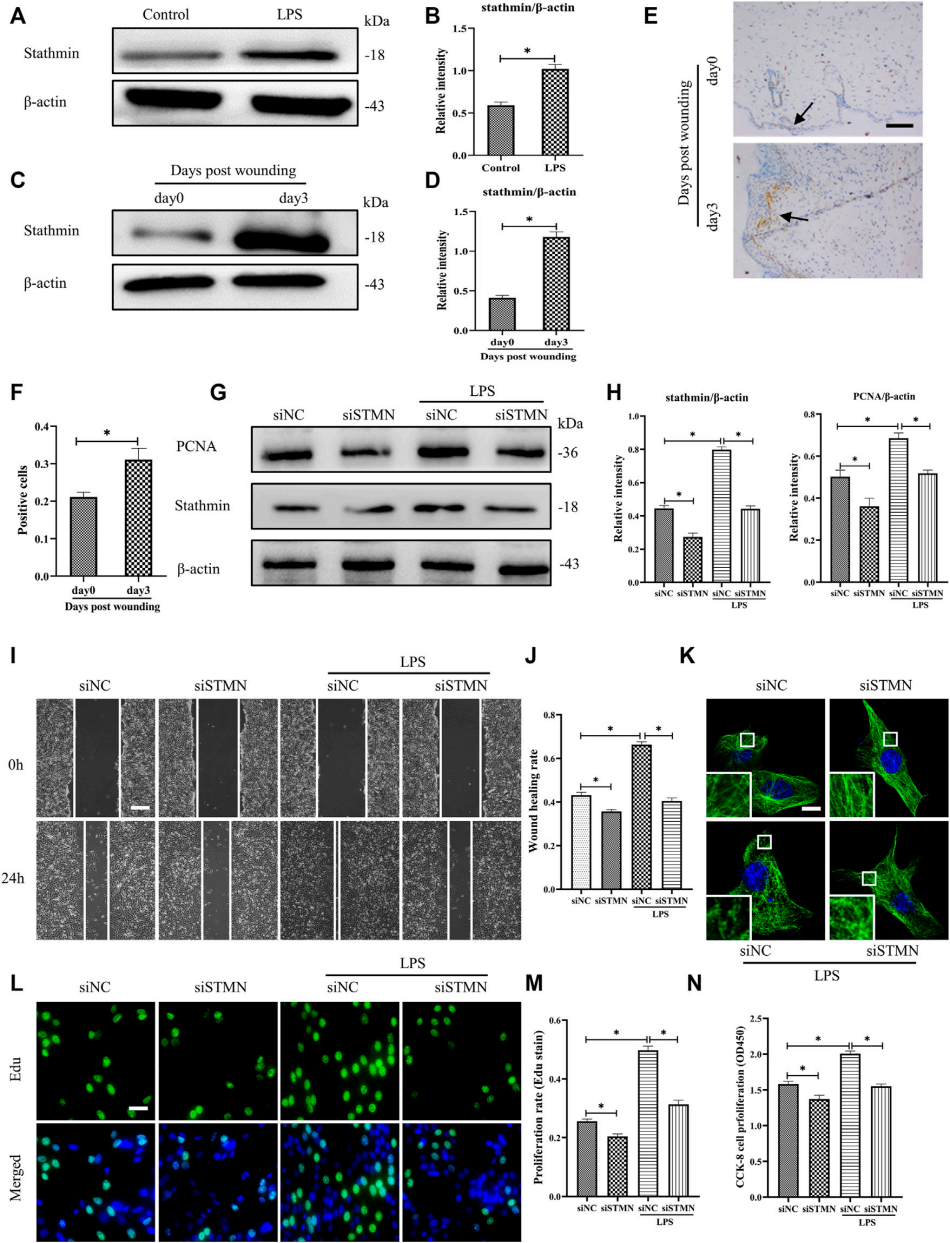
LPS-induced stathmin expression and its effects on HDF migration and proliferation and MT depolymerization. **(A)** Western blotting was performed to detect stathmin expression with or without LPS, and **(B)** the results were quantitatively analyzed (*n* = 5). **(C)** Western blotting was performed to detect stathmin expression in wound edge tissue post wounding, and **(D)** the results were quantitatively analyzed (*n* = 5). **(E)** Stathmin expression at the wound edge of mice post wounding was detected by immunohistochemical staining. Bar, 100 μm, the arrow points to the positive cells, and **(F)** the results were quantitatively analyzed (*n* = 5). **(G)** Cells were transfected with siSTMN or siNC before treatment with or without LPS stimulation. Western blotting was performed to detect stathmin and PCNA expression, and **(H)** the results were quantitatively analyzed (*n* = 5). **(I)** Scratch wound healing assays were performed to detect HDF migration treated with LPS after siSTMN or siNC transfection., Bar, 200 μm, and **(J)** the results were quantitatively analyzed (*n* = 5). **(K)** For the tubulin immunofluorescence images of MTs, cells were transfected with siNC or siSTMN before LPS treatment. The inserts show high-magnification images of the peripheral MT network. Bar, 10 µm. **(L)** The cells were transfected with siSTMN or siNC before LPS stimulation and detected by Edu staining, and **(M)** the results were quantitatively analyzed (*n* = 5). Nuclei were stained with Hoechst 33342. The merged image is to show the proportion of proliferating cells (green) to total cells (blue). Bar, 20 µm. **(N)** HDF proliferation was tested using the CCK-8 assay (*n* = 10). siSTMN, small interfering RNA for stathmin; siNC, small interfering RNA for negative control. The results are shown as means ± SEM. * *p* < 0.05 was considered to be significant.

To determine whether the above-noted MT depolymerization was attributed to stathmin expression changes, we first detected stathmin expression with LPS stimulation or in a vivo wound healing model (Figures 3A–F). Stathmin was found to show weak basal levels in cultured HDF, and expression of stathmin significantly increased after LPS stimulation (Figures 3A,B). In an *in vivo* model, a higher stathmin level at 3 days post wounding was noted compared to the control group, as represented by western blotting assay and immunohistochemistry staining (Figures 3C–F).

We then considered the possibility that stathmin was essential in mediating HDF migration and proliferation and MT depolymerization after LPS challenge. After transfection with siSTMN, the expression of stathmin decreased. We found that stathmin and PCNA expression levels were robustly elevated after LPS stimulation; however, after transfection with siSTMN, the expression levels of stathmin and PCNA were significantly decreased with or without LPS stimulation compared to their corresponding control groups (Figures 3G,H). A reduction in cell migration was observed after siSTMN transfection with or without LPS stimulation, as indicated by the scratch wound healing assay (Figures 3I,J). The siSTMN transfection abrogated cell proliferation with or without LPS stimulation (Figures 3L–N). A preserved MT network and an increased density of MT fragments were observed after siSTMN transfection with or without LPS stimulation (Figure 3K). Collectively, these data suggested that stathmin is a crucial regulator of HDF migration and proliferation and MT depolymerization during wound healing both in the absence and presence of LPS.

### P38/MAPK Activation Mediates Stathmin Expression in Lipopolysaccharide-Induced Human Dermal Fibroblast Migration and Proliferation

**FIGURE 4:**
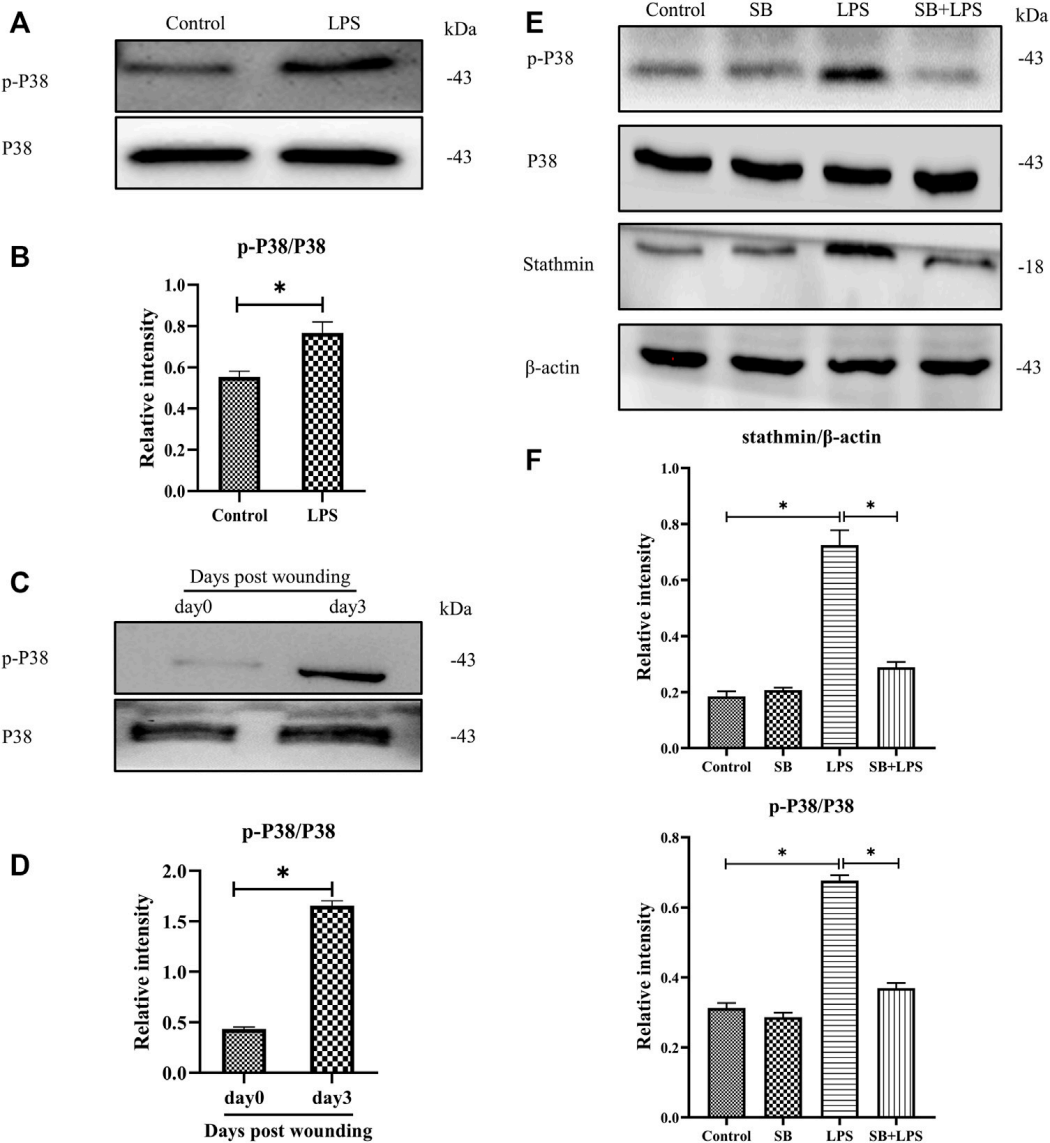
P38/MAPK activation mediates stathmin expression in LPS-induced HDF migration and proliferation. **(A)** Western blotting was performed to detect p38/MAPK activation with or without LPS, and **(B)** the results were quantitatively analyzed (*n* = 5). **(C)** Western blotting was performed to detect p38/MAPK activation in wound edge tissue post wounding, and **(D)** the results were quantitatively analyzed (*n* = 5). **(E)** Western blotting was performed to detect stathmin expression and p38/MAPK activation after LPS treatment with or without the p38/MAPK inhibitor SB, and **(F)** the results were quantitatively analyzed (*n* = 5). SB, SB203580. These data are represented as the mean ± SEM. ^*^ *p* < 0.05 was considered to be significant.

Previous studies have shown that p38/MAPK is critically important in fibroblasts (Molkentin et al., 2017). Besides, some other studies demonstrated that both p38 and ERK coordinate the dynamics of wound healing: while growth factor-stimulated p38 induced epithelial migration, and ERK1/2 activation induced cell proliferation (Sharma et al., 2003; Li et al., 2019). In our current study, to investigate whether p38/MAPK was involved in LPS-induced stathmin expression, MT depolymerization and HDF migration and proliferation, we first detected p38/MAPK activation. The results suggested that p38/MAPK was activated after LPS compared to the control group (Figures 4A,B). In addition, activated p38/MAPK was also observed at 3 days post wounding in animal studies compared to the control group (Figures 4C,D). We then tested whether the p38/MAPK signaling pathway was essential for stathmin regulation in LPS-induced HDF migration and proliferation. A p38/MAPK inhibitor, SB203580, was used in an *in vitro* study. We found that stathmin expression increased concomitantly with p38/MAPK activation by LPS stimulation of HDF, while SB203580 pretreatment showed comparable phosphorylated p38 and stathmin expression to the corresponding control group (Figures 4E,F). Conversely, pretreatment with the p38/MAPK inhibitor SB203580 significantly suppressed p38/MAPK activation and stathmin expression compared to the LPS group (Figures 4E,F). Thus, these results suggested that the p38/MAPK signaling pathway was responsible for stathmin regulation in LPS-induced HDF migration and proliferation.

### Role of p38/MAPK Activation in Lipopolysaccharide-Induced Human Dermal Fibroblast Migration and Proliferation and Microtubule Depolymerization

**FIGURE 5:**
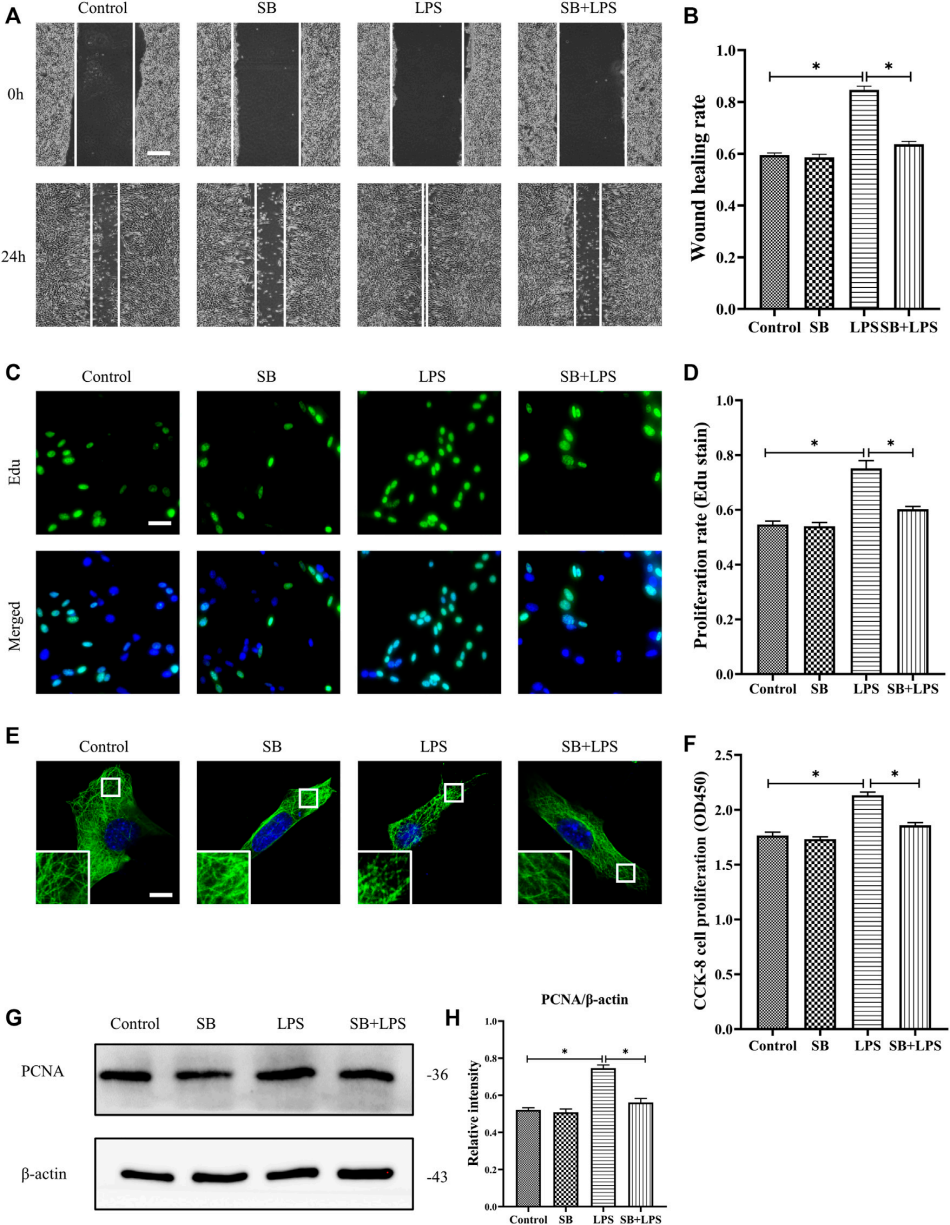
Role of p38/MAPK activation in LPS-induced HDF migration and proliferation and MT depolymerization. **(A)** Scratch wound healing assays were performed to detect HDF migration treated with LPS with or without SB (5 µm) pretreatment. Bar, 200 μm, and **(B)** the results were quantitatively analyzed (*n* = 5). **(C)** HDF proliferation was detected after LPS treatment with or without SB pretreatment by Edu staining, and **(D)** the results were quantitatively analyzed (*n* = 5). Nuclei were stained with Hoechst 33342. The merged image is to show the proportion of proliferating cells (green) to total cells (blue). Bar, 20 µm. **(E)** For the tubulin immunofluorescence images of MTs, cells were pretreated with SB before LPS treatment. These inserts show high-magnification images of the peripheral MT network. Bar, 10 µm. **(F)** HDF was subjected to LPS treatment with or without SB pretreatment to detect cell proliferation using the CCK-8 assay (*n* = 10). **(G)** Western blotting was performed to detect PCNA expression after LPS treatment with or without SB, and **(H)** the results were quantitatively analyzed (*n* = 5). The data are represented as the mean ± SEM. * *p* < 0.05 was considered to be significant.

We explored the role of p38/MAPK in the regulation of HDF migration and proliferation and MT depolymerization. The results reveal that LPS promoted HDF migration, as indicated by the scratch wound healing assay, whereas the application of the p38/MAPK inhibitor SB203580 largely abolished this effect (Figures 5A,B). Additionally, HDF proliferation was elevated after LPS challenge compared to the control group (Figures 5C,D,F–H). In contrast, SB203580 pretreatment suppressed the increased cell proliferation with LPS challenge and revealed a comparable change to the control group (Figures 5C,D,F–H). Concomitant with the suppression of HDF migration and proliferation, SB203580 preserved the MT network in the LPS-induced HDF and showed a similar MT structure compared to the control group (Figure 5E).

**FIGURE 6:**
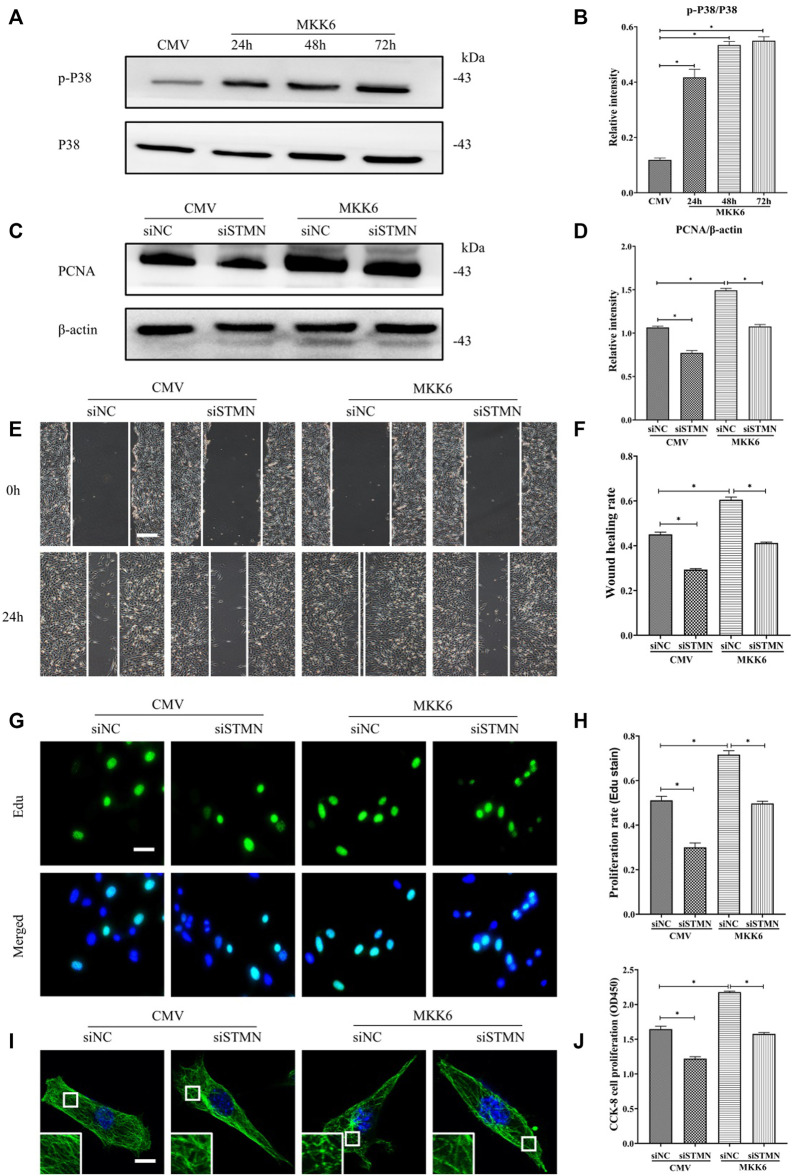
P38/MAPK induced HDF migration and proliferation *via* stathmin-dependent MT depolymerization. **(A)** Western blotting was performed to analyze the effects of MKK6(Glu) transfection for the indicated times, and **(B)** the results were quantitatively analyzed (*n* = 3). **(C)** Western blotting was performed to detect PCNA expression after the cells were transfected or cotransfected with siSTMN or MKK6(Glu), and **(D)** the results were quantitatively analyzed (*n* = 5). **(E)** Scratch wound healing assays were performed to detect HDF migration after cells were transfected or cotransfected with siSTMN or MKK6(Glu). Bar, 200 μm, and **(F)** the results were quantitatively analyzed (*n* = 5). **(G)** HDF proliferation was detected after the cells were transfected or cotransfected with siSTMN or MKK6(Glu) by EdU staining, and **(H)** the results were quantitatively analyzed (*n* = 5). Nuclei were stained with Hoechst 33342. The merged image is to show the proportion of proliferating cells (green) to total cells (blue). Bar, 20 µm. **(I)** For the tubulin immunofluorescence images of MTs, cells were transfected or cotransfected with siSTMN or MKK6(Glu). These inserts show high-magnification images of the peripheral MT network. Bar, 10 µm. **(J)** Cell proliferation was detected after the HDF was transfected or cotransfected with siSTMN or MKK6(Glu) using the CCK-8 assay (*n* = 10). The results are shown as the means ± SEM. * *p* < 0.05 was considered to be significant.

Then, we assessed the critical role of stathmin in mediating the p38/MAPK regulation of MT depolymerization and HDF migration and proliferation (Figure 6). A constitutively activated p38/MAPK activator, MKK6(Glu), was constructed and transfected into HDF to activate the p38/MAPK pathway (Figures 6A,B). MKK6 (Glu) overexpression promoted HDF proliferation, as represented by an increase in the proliferation marker PCNA (Figures 6C,D). In addition, elevated HDF proliferation was detected by Edu staining (Figures 6G,H) and CCK-8 kit testing (Figure 6J) after MKK6 (Glu) transfection. Conversely, transfection with siSTMN suppressed HDF proliferation with or without MKK6(Glu) compared to their corresponding control treatments (Figures 6C,D,G,H,J). In addition, MKK6(Glu) overexpression promoted HDF migration, as shown by a scratch wound healing assay, compared with the control group; in contrast, siSTMN transfection abrogated this effect with or without MKK6(Glu) overexpression (Figures 6E,F). Moreover, the HDF transfected with siSTMN were much more resistant than the control group to MT depolymerization in response to MKK6(Glu) transfection, as indicated by MT staining (Figure 6I). These findings are consistent with our concept of an essential role for stathmin in LPS-stimulated p38/MAPK-mediated MT depolymerization and HDF migration and proliferation.

## Discussion

**FIGURE 7:**
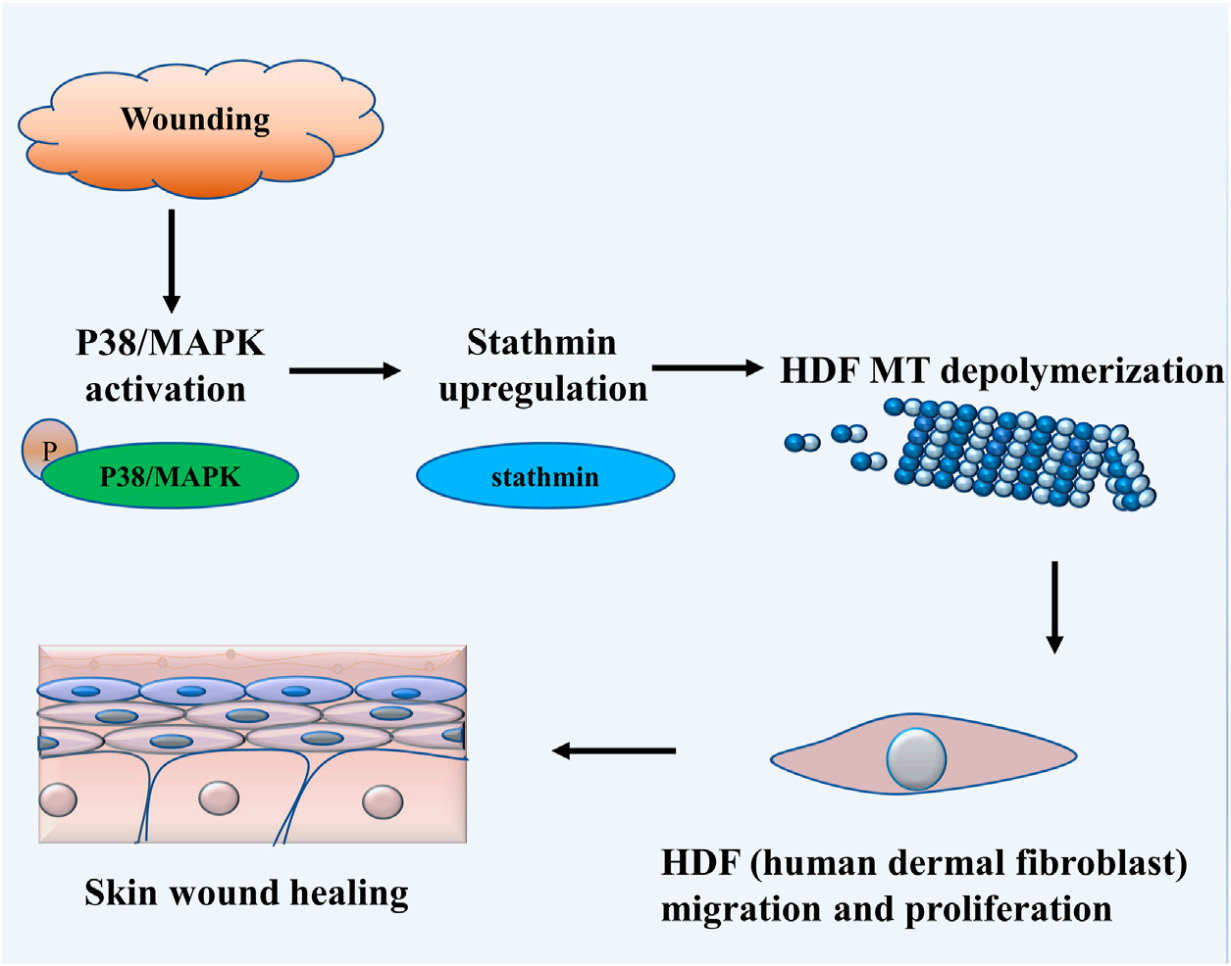
Schematic illustrating that stathmin promotes HDF migration and proliferation during wound healing. The LPS stimulates p38/MAPK activation and subsequently induces increased stathmin expression. Increased stathmin leads to MT depolymerization, which is required for HDF migration and proliferation, followed by skin wound healing.

The salient findings from our current study indicated that stathmin, a classical cytosolic MT-destabilizing protein, promoted HDF migration and proliferation, followed by wound healing. This notion was proved in both cell and animal models. Our data revealed that stathmin is elevated following wounding *in vivo*, as well as LPS exposure *in vitro* during a short time period, and is an important factor for wound healing. Stathmin increased concomitantly with p38/MAPK signaling activation by LPS stimulation of HDF, which led to MT depolymerization, accelerated HDF migration and proliferation, and the onset and progression of wound healing (Figure 7).

Wound healing is a complex and precisely regulated biological processes. Upon wounding, a large number of genes, including cytokines, chemokines, and antimicrobial peptides, are activated or expressed to initiate wound repair (Canedo-Dorantes and Canedo-Ayala, 2019; Rodrigues et al., 2019; Singampalli et al., 2020). Although the LPS exposure has been considered to be a crucial initial factor in wound healing (Ninan et al., 2015; Visan, 2019) and fibroblasts are deemed to play an essential role in this process (Desjardins-Park et al., 2018; Visan, 2019), the relationship and mechanisms between fibroblasts and LPS exposure remain less well defined. In our present study, we used LPS (500 ng/ml, 24 h) to mimic inflammatory stimulation *in vitro* during wound healing with a superimposed infection, because appropriate LPS stimulation could activate inflammatory response and start wound healing (Landén et al., 2016; Li et al., 2017), whereas inappropriate dose or treatment time of LPS may not promote wound healing, or even delay wound healing due to excessive oxidative stress or severe infection (Ning et al., 2021). We found that LPS induced elevated stathmin expression and promoted HDF migration and proliferation. Additionally, stathmin is robustly increased in wound edge tissue in animal models. Stathmin belongs to the stathmin family, which includes stathmin, SCG-10, SCLIP and RB3, and is ubiquitously expressed in various cell types (Cassimeris, 2002). It is a classical MT-depolymerization protein and involves a wide range of physiological and cellular functions (e.g., cell cycle, proliferation and migration) (Shu et al., 2019). However, whether stathmin is involved in LPS-induced HDF migration and proliferation is still unknown. Our results showed that stathmin was involved in LPS-mediated HDF migration and proliferation during wound healing.

The MT is a vital component of the cytoskeleton and involves a series of cellular functions, including organelle transport, cell cycle regulation, and maintenance of cellular morphology (Kapitein and Hoogenraad, 2015). Accumulating evidence has indicated that microfilament, another cytoskeleton component, is crucial in regulating cell migration, but whether MT is also involved in cell migration and even in cell proliferation should be further elucidated. The balance between MT depolymerization and polymerization reflects MT dynamics, which is affected by the factors that stabilize MTs, including MAPs, and the opposing effect that destabilizes MTs, such as the stathmin family (Mizumura et al., 2006). Previous studies suggested that stathmin is involved in tumor cell proliferation and invasion (Wang et al., 2016; Shu et al., 2019). However, whether stathmin is involved in HDF migration and proliferation during wound healing is uncertain. In our present study, we demonstrated that stathmin promoted HDF migration and proliferation upon wounding or LPS exposure. In addition, altered stathmin expression promoted MT dynamics changes, affecting the cell cycle, followed by cell proliferation (Rubin and Atweh, 2004), and MT depolymerization accelerated the assembly of focal adhesions and increased cell migration (Seetharaman and Etienne-Manneville, 2020), which supported our current findings. Thus, these data provide evidence of MT depolymerization in stathmin-mediated wound healing.

Our earlier studies suggested that stathmin-mediated MT depolymerization is involved in HDF migration and proliferation; however, the precise mechanism by which stathmin regulates MT dynamics deserves further elucidation. Accordingly, we demonstrated that p38/MAPK was activated concomitantly with elevated stathmin expression and MT depolymerization, meanwhile, phosphorylated p38/MAPK was elevated in wound edge tissue in animal models. In addition, growing evidence has revealed that p38/MAPK is a classical signaling pathway involved in wound healing and is recognized as a crucial pathway in regulating MT dynamics (Fabris et al., 2015; Esnault et al., 2020). Moreover, several studies have reported that the MAPK, MARK and PKA pathways are involved in the regulation of stathmin (Yip et al., 2014; Feng et al., 2017). We suppressed p38/MAPK under LPS stimulation. Decreased stathmin expression and MT depolymerization were observed, followed by reduced HDF migration and proliferation. In contrast, the transfection of MKK6 (Glu), an activator of p38/MAPK, could promote stathmin expression, MT depolymerization, and HDF migration and proliferation. Moreover, the application of siSTMN in HDF significantly abrogated the effect caused by MKK6 (Glu) transfection. Collectively, these data indicated that the p38/MAPK signaling pathway is an upstream kinase of stathmin, and is crucial for LPS-induced HDF migration and proliferation *via* stathmin-mediated MT depolymerization. In spite of this, it should be noted that several reports (Henklova et al., 2008; Muniyappa and Das, 2008) found that SB203580 could also activate ERK and JNK, both of which showed a certain role in migration and proliferation. Thus, we cannot completely exclude the role of ERK and JNK in our study, and further experiment would be shown to demonstrate the unique role of p38/MAPK in HDF migration and proliferation. Pathologic tissue repair usually refers to two types of wound healing: excessive healing and deficient healing. The excessive healing, such as keloids and hypertrophic scars, and the deficient healing, including diabetic foot ulcers and venous leg ulcers, are characterized by excessive and suppressed fibroblast proliferation respectively (Zou et al., 2021). In addition, p38/MAPK activation is deemed important in keloids (Song et al., 2012) and p38/MAPK inactivation is correlated with diabetic foot ulcers (Singh et al., 2015), indicating that p38/MAPK-mediated fibroblast proliferation is crucial in skin wound healing. In addition to its role in healing of skin wounds, stathmin and p38/MAPK are involved in tumor cells migration and proliferation (Yurong et al., 2017). Moreover, fibroblast overexpressing p38/MAPK induced interstitial and perivascular fibrosis in the heart, lung, and kidney (Molkentin et al., 2017). Considering a possible role for the interaction of p38/MAPK and stathmin in the current and previous studies, this interaction may be important in various hyper- and dysproliferative disorders. New studies are needed to address this possibility.

Taken together, the findings of our present study reveal a novel role of stathmin in HDF migration and proliferation during wound healing, while p38/MAPK serves as the upstream kinase of stathmin-mediated MT depolymerization, followed by HDF migration and proliferation. Furthermore, our results point to stathmin regulation as a potential target for the development of therapeutic interventions for wound healing.

## Data Availability

The original contributions presented in the study are included in the article/Supplementary Material, further inquiries can be directed to the corresponding authors.
